# Mixed Meal Tolerance Test Versus Continuous Glucose Monitoring for an Effective Diagnosis of Persistent Post-Bariatric Hypoglycemia

**DOI:** 10.3390/jcm12134295

**Published:** 2023-06-27

**Authors:** Ana M. Ramos-Levi, Miguel A. Rubio-Herrera, Pilar Matía-Martín, Natalia Pérez-Ferre, Clara Marcuello, Andrés Sánchez-Pernaute, Antonio J. Torres-García, Alfonso L. Calle-Pascual

**Affiliations:** 1 Departament of Endocrinology and Nutrition, Hospital La Princesa, Instituto de Investigación Princesa, Universidad Autónoma de Madrid, 28049 Madrid, Spain; ana_ramoslevi@hotmail.com; 2 Departament of Endocrinology and Nutrition, Hospital Clínico San Carlos, IdISSC, 28040 Madrid, Spain; 3 Faculty of Medicine, Department of Medicine, Universidad Complutense, 28040 Madrid, Spain; 4 Department of Surgery, Hospital Clínico San Carlos, IdISSC, Faculty of Medicine, Universidad Complutense, 28040 Madrid, Spain; 5 Centro de Investigación Biomédica en Red de Diabetes y Enfermedades Metabólicas Asociadas (CIBERDEM), 28040 Madrid, Spain

**Keywords:** gastric bypass, post-bariatric hypoglycemia, mixed meal tolerance test, continuous glucose monitoring

## Abstract

Gastric bypass determines an increase in incretin secretion and glucose excursions throughout the day and may sometimes entail the development of severe post-bariatric hypoglycemia (PBH). However, there is no consensus on the gold standard method for its diagnosis. In this study, we evaluated the usefulness of a mixed meal tolerance test (MMTT) and continuous glucose monitoring (CGM) for the diagnosis of PBH, defined as glucose levels <54 mg/dL (3.0 mmol/L). We found that hypoglycemia occurred in 60% of patients after the MMTT and in 75% during CGM, and it was predominantly asymptomatic. The MMTT confirmed the diagnosis of PBH in 88.9%of patients in whom surgery had been performed more than three years ago, in comparison to 36.4% in cases with a shorter postsurgical duration. CGM diagnosed nocturnal asymptomatic hypoglycemia in 70% of patients, and daytime postprandial hypoglycemia in 25% of cases. The mean duration of asymptomatic hypoglycemia was more than 30 min a day. Patients with ≥2% of their CGM readings with hypoglycemia exhibited a higher degree of glucose variability than those with <1% of the time in hypoglycemia. Our results show that the MMTT may be a useful dynamic test to confirm the occurrence of hypoglycemia in a large number of patients with persistent and recurrent PBH during long-term follow-up after gastric bypass. CGM, on its part, helps identify hypoglycemia in the real-world setting, especially nocturnal asymptomatic hypoglycemia, bringing to light that PBH is not always postprandial.

## 1. Introduction

Bariatric surgery (BS) has proven to be a very useful tool for the management of severe obesity since it allows significant long-term weight loss and amelioration, or even resolution of associated comorbidities [1]. Despite its well-known beneficial effects in improving patients’ metabolic syndrome and quality of life, BS also entails several controversies, especially regarding weight regain and reappearance of comorbidities [2], an unexplained increase in other causes of mortality [3], and deterioration of bone health [4]. 

Post-bariatric hypoglycemia (PBH) is one of the most defying challenges that patients and clinicians encounter during the follow-up after BS, but its physiopathology and diagnosis have not been fully established. PBH is characterized by the development of hypoglycemia, usually between one and three hours after a meal, with adrenergic and neuroglucopenic symptoms that improve after the administration of rapidly absorbed carbohydrates (Whipple’s triad) [5]. In the literature, this clinical picture has been usually referred to as postprandial hyperinsulinemic hypoglycemia (PHH); but given the frequent occurrence of hypoglycemia not related to a prior meal intake, especially during the night, the term PBH is generally preferred. Several studies have described an increased prevalence two years after Roux-en-Y gastric bypass, but it has also been reported in patients who underwent other types of procedures, such as sleeve gastrectomy, and one anastomosis gastric bypass [6].

There is no generalized consensus on how to effectively diagnose PBH [5]. Indeed, there is wide heterogeneity in the results observed across different studies, mainly due to the specific characteristics of patients included, the diagnostic methods, and the threshold values to define PBH. As a result, the prevalence of PBH has not been consistently established, since it is clearly dependent on the diagnostic method used to identify it. For instance, severe PBH, with associated neuroglucopenic symptoms, has been reported to occur in less than 1% of patients [7,8]. However, its prevalence may grow up to 30% of patients when specific questionnaires are used, [9] and more than 50% when dynamic tests such as an oral glucose tolerance test (OGTT) or a mixed meal tolerance test (MMTT) are used [5]. 

In an attempt to overcome the associated diagnostic difficulties, and with the availability of new technologies, continuous glucose monitoring (CGM) has been recently used to better evaluate glucose excursions in post-bariatric patients. These devices are easily placed on the patient, well tolerated, easily adjustable, and provide useful information regarding interstitial glucose values and glucose variability throughout the day and night, for several days. In fact, CGM aids in a deeper evaluation of glucose values, because it allows correlation in the setting of the patient’s specific lifestyle, for instance, times of meal intake, exercise, etc., and even helps detect asymptomatic periods of hypoglycemia. Thus, overall, it allows a more precise diagnosis of PBH. In addition, CGM may be useful to evaluate the effectiveness of specific dietary or pharmacological treatments [8]. 

The objective of this study is to evaluate the prevalence of PBH in a series of consecutive patients who underwent RYBG and who report recurrent postprandial hypoglycemia, using the MMTT and CGM for seven days. 

## 2. Materials and Methods

### 2.1. Participants

We performed a cross-sectional study with 20 patients without known diabetes, aged 23–65 years old, who had undergone bariatric surgery (BS) (specifically, Roux-en-Y gastric bypass) during the period between 2014 and 2019, and who referred hypoglycemic symptoms, according to Whipple’s triad. We collected data from their clinical records, including age, sex, body mass index (BMI) before BS and at the time of evaluation, time since BS, and percentage weight loss. Patients were excluded if they were taking any antidiabetic medications since surgery or if they had glycated hemoglobin (HbA1c) levels >6.0% or fasting blood glucose >100 mg/dL.

All patients signed a written informed consent. The study was approved by the Ethics Committee of the Hospital Clinico San Carlos (code CI-11/080E, approved on 21 September 2011), and was in compliance with the Helsinki Declaration. 

### 2.2. Mixed Meal Tolerance Test (MMTT)

Patients underwent a mixed meal tolerance test (MMTT) after a 12 h overnight fast. A peripheral venous catheterization was performed in the forearm to draw repeated blood samples at times 0, 30, 60, 90, 120, 180, and 240 min after intake of the standard beverage. Glucose and insulin levels were analyzed at these different times. The standard product used for the MMTT was TDiet 2.0^®^ (Vegenat Healthcare, Badajoz, Spain), which is a 200 mL 400 kcal beverage containing 45 g of carbohydrates, 20 g of proteins, and 15.5 g of total fat, and was taken in less than 10 min. 

### 2.3. Continuous Glucose Monitoring (CGM)

A CGM device was placed, after an overnight fast, for seven days, with surveillance of glucose levels in interstitial fluid every five minutes, 288 times per day (Medtronic Ipro2, Medtronic, Northridge, CA, USA). Each participant was instructed to calibrate their CGM device at least twice daily. A download of glucose data was performed using Carelink. Glucose variability (GV) was analyzed using standard measures of amplitude and timing, i.e., using mean, median, standard deviation (SD), variation coefficient (CV), minimum, maximum, and percentage of time under target ranges (<54 mg/dL, between 55 and 140 mg/dL, and >140 mg/dL) [10]. 

The device was placed on the patient by a trained nurse in the outpatient diabetes clinic. Each patient received comprehensive instructions regarding calibration of the CGM device, following the results of conventional capillary glucose monitoring, and according to the manufacturer’s instructions. Specifically, patients were trained to perform 4 capillary glucose tests throughout the day, three before each of the three main meals, and one before bedtime, according to the Ipro2 user manual. In addition, patients were asked to perform an additional capillary blood test at night if they woke up by any chance. The trained nurse ensured that instructions were properly understood. All patients performed finger-prick measurements with a glucometer, starting at 12 h after insertion of the CGM device, and every six hours thereafter, during the seven days of the study.

CGM devices provided information regarding the time in which the patient underwent a hypoglycemic event (blood glucose levels <54 mg/dL [3 mmol/L]) for more than 15 min. We collected data on (a) the number of diurnal postprandial hypoglycemic events, occurring between 06:00 and 00:00, after four hours from the last food intake, and (b) the number of nocturnal hypoglycemic events, occurring between 00:00 and 06:00, after four hours from the last meal. The following measurements were recorded: mean interstitial glucose (IG) (mg/dL), mean IG peak (mg(dL), mean IG nadir (mg/dL), standard deviation [SD (mg/dL)], and variation coefficient [CV (%)], percentage of time spent with IG <54 mg/dL, % of the time with IG 55–70 mg/dL, % of the time with IG 71–140 mg/dL and % of the time with IG >140 mg/dl.

Measurements were not obtained at the same time as the MMTT but were not delayed more than a couple of days. The MMTT was performed in the first place, and then, the CGM device was placed, with a time-lapse of just two days.

### 2.4. Dietary Intake during CGM

To estimate patients’ dietary intake, we used a self-reported record of seven consecutive days, which included at least one non-working day. Data were standardized using the EASY-DIET™ software (https://www.easydiet.es), from the Spanish Academy of Nutrition and Diet. Physical activity was quantified using the International Physical Activity Questionnaire (IPAQ) as METs/min/week.

### 2.5. Laboratory Tests

Plasma glucose levels were measured using the glucose oxidase method. HbA1c was analyzed with a method standardized by the International Federation of Clinical Chemistry and Laboratory Medicine, using ion-exchange high-performance liquid chromatography in gradient, with a Tosoh G8 analyzer (Tosoh Co., Tokyo, Japan). Serum insulin was measured by a chemiluminescence immunoassay in an IMMULITE 2000 Xpi (Siemens, Healthcare Diagnostics, Munich, Germany).

### 2.6. Statistical Analysis

Continuous variables were summarized as mean ± standard deviation. Categorical variables were expressed as percentages. The incremental area under the curve (iAUC) was calculated using the trapezoidal rule, with the deduction of fasting hormonal levels from subsequent time points.

Comparison between continuous variables was performed using an independent-sample *t*-test. For variables with a skewed distribution, Mann Whitney U-test was used for mean comparisons. The Chi-square test was used to analyze categorical data. Linear regression analysis was used to adjust for potential confounders identified in univariate analysis. Repeated measures ANOVA was conducted for each outcome using “time” (for each of the measurements performed at different times) as a within-subjects factor and “group” (total severe hypoglycemia <1% vs. total severe hypoglycemia >2%) as a between-subjects factor. All statistical analysis was performed using JASP Team (2023, version 0.17.1 computer software).

## 3. Results

Eighteen women and two men without previously diagnosed diabetes, who had undergone Roux-en-Y gastric bypass and reported repeated postprandial hypoglycemic symptoms, were included in the study. The mean age was 43.0 ± 10.5 years, and the mean presurgical BMI was 43.9 ± 7.1 kg/m^2^. At the time of evaluation, the median time from BS was 24 months (IQR 21–51), and the mean BMI was 28.5 ± 3.9 kg/m^2^, with a percentage weight loss of 34.2 ± 8.7. 

Symptoms of hypoglycemia occurred after one to three hours from the last food intake. The most frequently reported (>90%) symptoms were overall general weakness and dizziness. Tremors and palpitations were reported by 55% of patients. Other neuroglucopenic symptoms, such as sweating, blurred vision, or confusion, were not consistently reported. 

### 3.1. Mixed Meal Tolerance Test

Twelve patients (60%) presented hypoglycemia (glucose values <54 mg/dL [3.0 mmol]) [Hypo group]. Compared with the group without biochemical hypoglycemia (Non-Hypo), glycemia nadir was significantly lower (mean ± SD: 41.9 ± 8.4 mg/dL, for 90–180 min, vs. 70.9 ± 15.5 mg/dL; *p* < 0.001), following prior hyperinsulinemia (mean insulin level 206.2 ± 79.7 µUI/mL during the period of 30–60 min, in the hypo group, than in the non-Hypo group (108.2 ± 47.2 µUI/mL; *p* = 0.003). There were no differences between both groups in age, BMI, time since surgery, HbA1c, C-Peptide, HOMA-IR, and glycemic variability. Only four patients reported severe symptoms of hypoglycemia when their glucose values dropped below 40 mg/dL. When this occurred, the MMTT was stopped (min 90–120), hypoglycemia was treated by oral administration of 15 g of rapidly absorbed carbohydrate, and glucose levels were monitored every 15 min until values were safely above 60 mg/dL. The rest of the patients in the hypo group were either asymptomatic or had mild symptoms of hypoglycemia that did not require discontinuation of the test.

**Figure 1:**
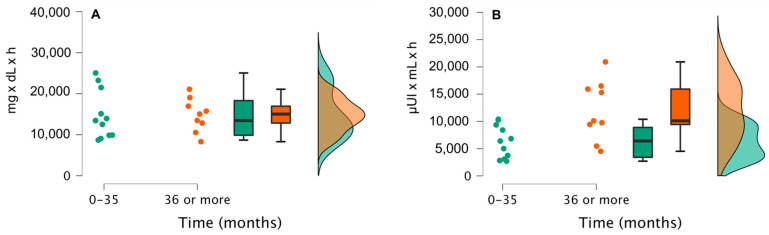
The graph shows glucose (**A**) and insulin (**B**) secretion profiles, according to the time elapsed after bariatric surgery. Individual glucose and insulin AUC values are shown, as well as the boxplot representing the AUC (median and interquartile range). Curves on the right show the results for the MMTT for subjects in whom surgery was performed before (green) or more than (orange) three years ago.

Patients who had undergone surgery more than 36 months before the study (group A) significantly exhibited more hypoglycemic episodes (glucose values < 54 mg/dL) after the MMTT than patients who had undergone surgery less than 36 months ago (group B) (88.9% vs. 36.4%, *p* = 0.0281). In addition, the former group had higher prior peak insulin levels (209.4 ± 82.5 vs. 129.9 ± 73.3 µUI/mL; *p* = 0.035). The area under the curve (AUC) was not significantly different for glucose values between both groups (14,778.3 ± 4021.2 mg × dL × min for group A and 14,754.6 ± 5900.9 mg × dL × min for group B), although differences were observed for the insulin AUC (11,986.7 ± 5476.4 vs. 6269.4 ± 3002.3 µUI × mL × h; *p* = 0.020 (Figure 1).

### 3.2. Continuous Glucose Monitoring (CGM)

The mean number of readings for a median of seven days was 1705.9 ± 204.4. Fifteen patients (75%) presented at least one hypoglycemic episode (glucose reading < 54 mg/dL) for more than 15 min, and 3.1% of the total number of glucose readings. Amongst patients with hypoglycemia, the mean number of episodes was 4.2 ± 2.7 and glucose values were between 54–70 mg/dL for 19.5% of the time. Eight patients (66.7%) had nocturnal hypoglycemia, with a mean number of 42.2 ± 2.7 events. This meant that nocturnal hypoglycemia was recorded in 2.5 ± 2.0% of the total amount of glucose readings, meaning 213.2 ± 188.6 min throughout the six days of CGM. 

Only five patients (25%) had diurnal postprandial hypoglycemia, with a mean number of 9.6 ± 30.4 episodes (3.2 ± 2.1% of the total number of readings and 198.0 ± 151.9 min). We did not find significant differences in the number of diurnal or nocturnal hypoglycemic events according to the time elapsed after BS. A median of 1% of CGM readings in hypoglycemia (<54 mg/dL) was considered as the cut-off point to define those subjects with a higher or lower percentage of hypoglycemia during the seven days of CGM recording. So, if patients with severe hypoglycemia in ≤1% of their readings (*n* = 11) were compared to those with ≥2% of their readings (*n* = 9), we observed that the latter group exhibited a greater amount of time in overall hypoglycemia, during both day and night and with a higher glucose variability (CV 0.26 ± 0.04 vs. 0.21 ± 0.05; *p* = 0.039) (Table 1). 

Multivariate analysis after adjusting for gender, age, presurgical BMI, actual BMI, weight loss, time since surgery, and peak insulin levels in the MMTT, and repeated measures ANOVA for time and group did not show significant results for their association with hypoglycemic events.

### 3.3. Dietary Intake during CGM

Total dietary intake was not significantly different between patients with fewer hypoglycemia events (<1%) in comparison to those with frequent PBH (≥2%): 969.8 ± 149.6 vs. 1016.1 ± 220.2 kcal; *p* = 0.583. Regarding the distribution of macronutrients, we observed that there was a significantly higher intake of protein (29.9 ± 3.8% vs. 24.6 ± 3.6%; *p* = 0.005), but not a significant difference in the intake of carbohydrates (34.0 ± 3.4% vs. 38.9 ± 11.2, *p* = 0.214) or fat (36.1 ± 3.0% vs. 38.2 ± 11.1%; *p* = 0.580) in patients who had frequent PBH (≥2%). Physical activity was also not different between patients with <1% or ≥2% hypoglycemia events during CGM: 1411.7 ± 845.1 vs. 1449.8 ± 1062.2 MET/min/wk; *p* = 0.945.

## 4. Discussion

Our study reveals that more than 50% of patients with prior BS and symptoms of PBH do indeed present hypoglycemia, with glucose levels below 54 mg/dL measured in an MMTT and CGM. Even though the clinical relevance and interpretation of hypoglycemia observed with these two diagnostic methods differ, it is a very significant finding, with implications in everyday clinical practice. Interestingly, in our cohort of patients, 60% experienced hypoglycemia after an MMTT, whilst only 20% referred symptoms at the time of glucose values below 40 mg/dL. Patients who experienced postprandial hypoglycemia after the MMTT (Hypo group) in our series had also a significant increase in prior insulin levels (>200 µUI/mL), which doubles levels of patients without postprandial hypoglycemia (non-hypo group). 

The underlying mechanism involved in the occurrence of PBH has been associated with the increased gastric emptying observed after Roux-en-Y gastric bypass, entailing an increased glucose absorption, which is accompanied by a significant increase in incretin levels, including GLP-1 [11,12,13]. In this regard, when GLP-1 is blocked by administration of its antagonist exendine 9–39, there is no hyper-insulinemic response, and hypoglycemia reverts in all patients exhibiting PBH [14]. An imbalance in the counter-regulatory response mediated by glucagon has also been suggested; specifically, lower early peak levels have been observed after MMTT in patients with hypoglycemia, which were unable to counter-regulate the over-elevated levels of insulin [12,13]. Even though this mitigated glucagon response has been consistently reported in several studies, some authors have not observed a significant difference in levels of patients with no PBH after RYGB [15], suggesting that this analytical finding may be inherent to altered post-bariatric surgery physiology, in the setting of sustained weight loss and lower nadir glucose levels, and not a true cause of PBH. Interestingly, in the aforementioned study, the authors found that levels of pancreatic polypeptide (PP) were significantly decreased in patients with PBH, in comparison to the control group. Given the fact that PP is a surrogate marker of parasympathetic input to the pancreas and a marker of autonomic hypoglycemia counter-regulation, in addition to glucagon and catecholamines, the authors speculated a global attenuation of neurohormonal responses to insulin-induced hypoglycemia in post-bariatric surgery patients experiencing substantial weight loss [15]. 

In our study, we observed a higher frequency of hypoglycemia in patients who had undergone BS more than three years ago, in agreement with previous reports [6,16]. A potential explanation for this finding may be related to a greater widening of the gastroyeyunal anastomosis over time, which would enable an increased gastric emptying and a greater incretin and insulin response to equivalent carbohydrate intake. In fact, in patients who underwent BS more than three years ago, peak insulin levels and AUC were much higher than in patients in whom surgery was performed less than 3 years ago.

We remark on the fact that the duration of the MMTT in our series was extended to 240 min because some patients may experience what is known as postprandial delayed hypoglycemia. Indeed, in three of the 12 patients in the Hypo group (25%), the minimum glucose levels were reached after 180 min. This consideration must be taken into account, especially in the specific setting of post-bariatric patients since shorter evaluations of only 120 min after intake of the mixed meal may underestimate the true prevalence of postprandial hypoglycemia during the MMTT and reduce its utility for diagnosis. This issue may turn markedly relevant. For instance, according to Halperin et al., CGM exhibits a higher sensibility and specificity (90% and 50%, respectively) for detecting hypoglycemia than MMTT (33% and 40%, respectively) [17]. Kefur et al. found similar trends favors to CGM [18]. However, Honka et al. presented opposite results; they found a higher sensibility and specificity (77% and 100%, respectively) for the MMT for detecting hypoglycemia [19]. It is worth noting that Halperin et al. [17] performed their MMTT with a mixed meal containing 40 g of carbohydrates, and a duration of only 120 min, which, as previously mentioned, may reduce the test’s sensibility. Kefur et al. [18], on their part, used a mixed meal with only 28 g of carbohydrates, which is not enough for detecting hypoglycemia, and, therefore, also reducing their test’s sensibility. Using around 50 g of carbohydrate mixed meal and a sufficiently long duration for the test, similar to the one performed in our study, the sensibility and specificity of the MMTT are significantly better than for CGM [6,19]. The composition of the MMTT may also be relevant, but it has not been consistently standardized. Although the total glucose load is lower than in the OGTT, the mixed intake of protein and fat also triggers insulin secretion. In addition, because the usual presentation of the mixed meal is in the format of a liquid preparation, it passes quickly through the gastric pouch and the small intestine, thereby potentially increasing the risk of early dumping and PBH. 

From a clinical point of view, CGM seems more attractive than an MMTT to detect hypoglycemia, since it allows the analysis of glucose excursions during day and night, for several days, in relation to real-world patient’s everyday life, and even detects asymptomatic and unawareness hypoglycemia, such as nocturnal hypoglycemia, not only postprandial hypoglycemia [20]. In a recent meta-analysis of eight studies including 280 post-bariatric patients, around 50% exhibited diurnal and nocturnal hypoglycemia, according to CGM. Therefore, the authors concluded that CGM is the most efficient and precise method for detecting any form of PBH [6], in agreement with previous studies [17,18]. Another interesting finding of this meta-analysis is that patients with previous RYGB exhibit a greater glucose variability than patients who underwent sleeve gastrectomy (SG); in fact, they noted that diurnal hypoglycemia was more characteristic for post-SG patients, whilst nocturnal hypoglycemia was more frequently observed in post-RYGB patients. In line with these observations, a previous study performed by our group revealed a greater glucose variability in patients with prior RYGB, in comparison to patients in whom their BS preserved the gastric pyloric sphincter [21]. 

In line with this previous point, when evaluated using CGM, our patients showed a higher frequency of hypoglycemia (75% of patients), with a clear nocturnal and asymptomatic predominance (70%, versus 25% postprandial). This means that post-RYGB patients experience a mean duration of at least 30 min with nocturnal asymptomatic severe hypoglycemia (<54 mg/dL). Unawareness of hypoglycemia may be due to repeated chronic hypoglycemia, which reduces the threshold for detection and triggering of counter-hormonal response [20], or aberrant regulation of glycogenolysis and/or neoglycogenesis during the night [22]. In this scenario, it is advisable to screen patients for nocturnal sweating, poor sleep quality, restless dreams, and morning headaches as potential symptoms linked to nocturnal hypoglycemia [22]. In this regard, for instance, our patients only reported symptoms of hypoglycemia after the MMTT when glucose levels were below 40 mg/dL. The clinical implication of this finding is highly relevant, since repeated unawareness of hypoglycemia may entail deleterious effects on cognitive function [23] and is associated with an increased risk of non-fatal stroke, cardiovascular-related death, and total mortality in patients with diabetes [24,25].

The few studies that evaluate dietary intake and physical activity during CGM have not been able to prove significant differences between patients with or without postprandial hypoglycemia. In fact, there have been no differences either in macronutrient distribution, i.e., in the intake of rapidly absorbed carbohydrates, glycemic index, or glycemic load [20,26]. In agreement with these previous reports, we have not found significant differences in overall dietary intake or physical activity in our cohort of patients, although a very subtle higher intake of protein in patients with frequent PBH. Additional studies are needed to elucidate the relationship between the quantity and quality of carbohydrates and proteins on insulin secretion in post-bariatric patients.

We did not find an association between the observed response of the Hypo and Non-hypo groups to the MMTT, and the frequency of overall hypoglycemia detected with CGM. However, when we stratified patients according to a low prevalence of hypoglycemia (≤1%) versus a high prevalence (≥2%), glucose variability was greater and pre-surgery BMI was lower in patients with a higher frequency of hypoglycemia, similar to what has been previously reported [27]. Accordingly, we did not find differences in glucose and insulin curves after the MMTT between both groups. Interestingly, 82% of the group with <1% hypoglycemia was adequately controlled with diet and alpha-glucosidase inhibitors within a few weeks, whereas 78% of the group with ≥2% hypoglycemia required 2 or more drugs to control hypoglycemia symptoms. Some patients even required endoscopic adjustment of gastro-jejunal anastomosis using argon plasma coagulation and/or the Apollo overstitch procedure. 

Therefore, we can assume that, from a clinical point of view, the MMTT and CGM retrieve different results in patients that refer to PBH, but these results may complement each other. In this regard, the MMTT is a provocative dynamic test that helps establish the confirmed diagnosis of PBH in 89% of our patients with a longer duration after BS but would be less predictive in patients reporting PBH with a shorter follow-up period after BS. On the other hand, CGM would serve as an alert for detecting any type of hypoglycemia (postprandial, nocturnal, asymptomatic, symptomatic), in any case, scenario of everyday life, regardless of the time elapsed after BS, the percentage weight loss or the result after the MMTT, confirming the usefulness and convenience of CGM in the real-world setting [6]. 

A debatable matter regarding CGM is the specific type of device that should be used and the threshold values for defining hypoglycemia. These devices usually measure glucose levels in the interstitial liquid of the subcutaneous adipose tissue and prove an acceptable correlation with plasma glucose levels. In our study, we defined hypoglycemia according to a Joint Position Statement of the American Diabetes Association and the European Association for the Study of Diabetes as glucose levels <54 mg/dL (<3.0 mmol/L), detected by self-monitoring of capillary glucose, continuous glucose monitoring or a laboratory measurement [28]. It is rare that this level is reached under physiological conditions in nondiabetic individuals. Moreover, this threshold was chosen according to the study by Shah et al., performed in healthy non-diabetic individuals wearing CGM for 10 days [29]. They found that 14% of participants had a hypoglycemic event overnight. Overall, 35% of participants spent ≥2% of the time with sensor glucose <70 mg/dL (almost 30 min/d), but only 1% of participants spent ≥2% of time <54 mg/dL. [28]. This could be viewed as the most accurate approximation to the definition of PBH and for decision-making regarding the treatment of critical situations, especially in the setting of patients with diabetes taking glucose-lowering drugs. 

However, discrimination of glucose levels in the range of severe hypoglycemia is quite poor for the majority of CGM devices, and this should be taken into account [30]. In fact, one of the most critical aspects of studies evaluating hypoglycemia concerns the accuracy and reliability of interstitial glucose measurements with different devices, which is performed using the mean absolute relative difference (MARD) between CGM readings and paired blood glucose values [31]. The ideal device would have a MARD < 10%, but the mean MARD for the majority of CGM devices is well above that target, and in the range of hypoglycemia, it may even be >20% [32,33]. The majority of studies evaluating PBH have used Medtronic or Dexcom devices, with similar results using different thresholds (<70 mg/dL, <60 mg/dL, or <50 mg/dL) [6]. Another important consideration is the location for placing the device, since there may be noteworthy reading errors if the device is trampled, for instance, if the patient rests on his/her arm, which is the usual site, presumably, due to local blood-flow decreases caused by tissue compression [34]. To avoid this potential confounder, our patients wore the device on the abdomen.

Our study has some limitations. First, we only evaluated patients with a prior history of repeated hypoglycemia after RYGB, without a control group of asymptomatic patients with the same type of BS, which may hinder the potential detection of differences in cases of asymptomatic hypoglycemia. Second, we were unable to perform detailed dietary and exercise recordings to account for all glucose excursions during CGM. Additionally, even though patients calibrated the device according to capillary glucose levels every six hours, calibration was withheld during the patients’ night-time rest. Thus, we lack a paired comparison of the accuracy of the correlation of CGM and capillary glucose levels during the periods in the range of hypoglycemia. In any case, we remark on the strengths of our study, which concern the performance of an MMTT with a full evaluation of glucose and insulin curves for 240 min, improved potential detection of hypoglycemia, and the concomitant use of a CGM device for seven days to evaluate all glucose excursions occurring during this period of time. 

## 5. Conclusions

In patients with recurrent symptoms of PBH after RYGB, the MMTT confirms the diagnosis of postprandial hyperinsulinemic hypoglycemia when bariatric surgery was performed more than three years ago. Detection of nocturnal asymptomatic hypoglycemia with CGM jeopardizes the idea that PBH is predominantly postprandial. Additional studies with accurate CGM devices in the range of hypoglycemia are needed to better explore PBH.

## Figures and Tables

**Table 1:** Clinical and laboratory characteristics after the mixed meal tolerance test (MMTT) and continuous glucose monitoring (CGM) in subjects with different duration in severe hypoglycemia (<54 mg/dL).

Total Severe Hypoglycemia	≤1%	≥2%	*p*
Age (years)	40.2 ± 6.7	46.4 ± 9.7	0.517
BMI at time of BS (kg/m^2^)	47.03 ± 7.1	39.9 ± 5.1	0.033
BMI at time of CGM (kg/m^2^)	29.9 ± 3.6	26.8 ± 3.8	0.119
%WL at time of CGM	35.4 ± 10.6	32.7 ± 5.9	0.412
Time from BS (months)	42.6 ± 23	24.1 ± 12.0	0.073
HbA1c (%) at time of CGM	5.56 ± 0.4	5.2 ± 0.4	0.113
C-peptide at time of CGM	1.73 ± 0.78	1.34 ± 0.24	0.227
HOMA-IR at time of CGM	1.24 ± 0.82	1.19 ± 0.72	0.893
AUC glycemia MMTT (mg × dL × min)	13,548.6 ± 3661.6	16,208.3 ± 6219.1	0.254
AUC insulin MMTT (µUI × mL × min)	9639.6 ± 4424.8	7878.5 ± 5121.8	0.453
Mean BG (mg/dL)	89.5 ± 13.9	75.1 ± 8.8	0.022
Max BG (mg/dL)	175.6 ± 44.1	160.9 ± 23.8	0.518
Min BG (mg/dL)	48.4 ± 5.4	40.4 ± 1.3	0.002
% time in BG <54 mg/dL	0.55 ± 0.52	6.33 ± 4.18	<0.001
% time in BG 55–70 mg/dL	16.7 ± 13.4	33.2 ± 15.4	0.015
% time in BG 71–140 mg/dL	78.1 ± 11.3	58.9 ± 11.3	0.009
% time in BG >140 mg/dL	3.6 ± 6.1	1.56 ± 1.51	0.876
SD (mg/dL)	19.5 ± 6.1	19.3 ± 3.7	0.958
CV (mg/dL)	0.215 ± 0.05	0.261 ± 0.04	0.039
Diurnal hypoglycemias (%)	0.05 ± 0.17	1.73 ± 2.31	0.026
Nocturnal hypoglycemias (%)	0.79 ± 0.49	3.41 ± 1.92	0.009
Nocturnal hypoglycemias (min)	65.0 ± 48.9	295.6 ± 187.8	0.009

BMI: body mass index. BS: bariatric surgery. WL: weight loss. AUC: area under curve. BG: blood glucose. SD: standard deviation. CV: variation coefficient. Multivariate analysis after adjusting for gender, age, presurgical BMI, actual BMI, weight loss, time since surgery, and peak insulin levels in the MMTT did not show significant results for their association with hypoglycemic events.

## Data Availability

Not applicable.
